# Effect of NCOR1 Mutations on Immune Microenvironment and Efficacy of Immune Checkpoint Inhibitors in Patient with Bladder Cancer

**DOI:** 10.3389/fimmu.2021.630773

**Published:** 2021-03-08

**Authors:** Anqi Lin, Zhengang Qiu, Jian Zhang, Peng Luo

**Affiliations:** ^1^Department of Oncology, Zhujiang Hospital, Southern Medical University, Guangzhou, China; ^2^Department of Oncology, First Affiliated Hospital of Gannan Medical University, Ganzhou, China

**Keywords:** nuclear receptor corepressor 1 (NCOR1), mutations, immune checkpoint inhibitor, bladder cancer, tumor microenvironment

## Abstract

Immune checkpoint blockade (ICB) therapy has significantly progressed the treatment of bladder cancer (BLCA). Multiple studies have suggested that specific genetic mutations may serve as immune biomarkers for ICB therapy. Additionally, the nuclear receptor corepressor 1 (NCOR1) gene is a new player in the field of immune tolerance and the development of immune cells. In the ICI-treated-cohort, NCOR1 mutations may be used as a biomarker to predict the prognosis of BLCA patients receiving ICIs. The overall survival (OS) of the NCOR1-mutant (NCOR1-MT) group was significantly longer than that of NCOR1-wild-type (NCOR1-WT) group (P = 0·031; HR [95%CI]: 0·25 [0·12–0·52]). In the TCGA-BLCA-cohort, compared with NCOR1-WT, NCOR1-MT was associated with known predictors of ICB therapy efficacy, such as higher tumor mutational burden (TMB), neoantigen load and the number of mutations in the DNA damage-repair pathway. In addition, NCOR1-MT tumors had highly infiltrating TILs, activated antitumor immunity, and a high expression of immune-related genes, suggesting that NCOR1 mutations may serve as a potential biomarker to guide ICB therapy in BLCA.

## Introduction

Bladder cancer is the ninth most common malignant tumor worldwide, with approximately 540,000 new cases and 180,000 deaths each year (1). Due to its high recurrence rate after treatment and its biological behavior of malignant progression, routine treatment (such as intravesical bacillus Calmette-Guerin or platinum-based chemotherapy) frequently fails (2). For example, the median overall survival (OS) in relapsed or refractory bladder cancer patients with cisplatin is only 14–15 months; thus, alternate treatment strategies are needed (3). In recent years, immune checkpoint blockade (ICB) therapies, especially anti-programmed cell death (ligand) 1 (anti-PD-(L)1) and anti-cytotoxic T lymphocyte-associated antigen 4 (anti-CTLA-4), have provided new hope for bladder cancer patients. The United States Food and Drug Administration has approved five PD-(L)1 inhibitors for use as second-line or first-line treatment for advanced bladder cancer (3). In advanced bladder cancer, the overall response rates for immune checkpoint inhibitors (ICIs) are 13–24% (4–6), but most patients do not benefit from ICIs. Therefore, screening biomarkers for immunotherapy is particularly important to predict treatment response.

In addition to tumor mutational burden (TMB), microsatellite instability, mismatch repair gene deficiency, T-cell inflamed and IFN-*γ* gene expression profiles (GEPs), and DNA damage response (DDR) and antigen presentation defects may serve as potential biomarkers for ICB therapy (7). Studies have shown a correlation between specific genetic mutations and the efficacy of immunotherapy (8–10). POLE mutations are not only associated with high TMB and highly expressed immune checkpoint genes but are also linked to more durable clinical benefit from ICIs (11). Truncated mutations in PBRM1 are associated with improved clinical outcomes in patients with metastatic renal clear cell carcinoma receiving ICIs (12). TET1, SERPINB3 or SERPINB4 mutations are associated with improved antitumor immune responses in different tumors (*e.g.*, pan-cancer and melanoma) (10). In addition, changes in the genome and epigenome are the main driving forces for the pathogenesis of bladder cancer, which suggests that specific genetic mutations may have potential value as biomarkers predicting treatment response in bladder cancer (3, 13).

Recently, research has suggested that nuclear receptor corepressor 1 (NCOR1) plays an essential role in immune tolerance and T cell development (14, 15). The deletion of NCOR1 leads to immune tolerance in dendritic cells (DCs), the differentiation trend of regulatory T cells (Tregs) and the upregulated expression of genes related to immune depletion (15). In addition, NCOR1-histone deacetylase (NCOR1-HDAC) complexes can inhibit Treg activity (14).

In this study, based on the ICI-treated bladder cancer cohort with mutation and immunotherapy prognosis data (reporter by Samstein et al.), we analyzed the efficacy of ICIs based on NCOR1 alteration status. The results indicated that NCOR1 mutations may be a potential biomarker for bladder cancer patients receiving ICIs. In addition to prolonged OS after treatment with ICIs, NCOR1 mutations were associated with higher tumor immunogenicity, activated antitumor immunity, a number of mutations in the DDR pathway, immune cells and upregulated expression of immune-related genes.

## Materials and Methods

### Identification of the Association Between NCOR1 Mutations and Prognosis of Immunotherapy

To assess the association between NCOR1 mutations and the prognosis of bladder cancer patients receiving ICIs, we collected an ICI-treated cohort [Samstein et al. (16)] with clinical and mutation data. With the clinical data after ICI therapies (targeting PD-1/PD-L1 and CTLA-4) and mutation data for cancer (n = 210), Kaplan–Meier (KM) analysis was performed for the ICI-treated cohort based on NCOR1 alteration status. The exclusion criteria for further analysis were samples with missing follow-up time or missing mutation data (n = 5). Data licensed under Creative Commons 3.0 from http://research-pub.gene.com/IMvigor210CoreBiologies/ were downloaded *via* the IMvigor210CoreBiologies package (17); the data included the genome, transcriptome, and corresponding clinical characteristics of patients with metastatic urothelial cancer (mUC) who received atezolizumab (an anti-PD-L1 agent).

### The Genomic Characteristics of NCOR1 and Tumor Immunogenicity Analysis

The mutation data of the bladder cancer samples reported by Samstein et al. were sequenced by targeted next-generation sequencing (NGS; MSK-IMPACT). The neoantigen load (NAL) data in The Cancer Genome Atlas Urothelial Bladder Carcinoma (TCGA-BLCA) cohort were obtained from a literature review (18). The mutation data of the TCGA-BLCA cohort were downloaded using the “TCGAbiolinks” package in R software (19). Consistent with other literature (20), non-synonymous mutations in TCGA-BLCA were used as raw mutation counts and divided by 38 Mb to quantify TMB. The “ComplexHeatmap (21)” R package was used to visualize the mutational (top 20 mutational rate genes) and clinical profiles of the ICI-treated and TCGA-BLCA cohorts. The R package “Maftools (22)” was used to visualize NCOR1 mutation sites.

### Tumor Microenvironment Analysis

The “TCGAbiolinks” R package was used to download the gene expression data (Illumina HiSeq, RNA-Seq) of the TCGA-BLCA cohort (19). In this study, the GEPs of the TCGA-BLCA cohort were analyzed using the CIBERSORT (23) algorithm and the LM22 gene set to infer 22 human immune cell type proportions. In addition, we compared the expression of immune-related genes between the NCOR1-wild-type (NCOR1-WT) and NCOR1-mutant (NCOR1-MT) patients in the TCGA-BLCA cohort. The immune-related gene sets, functional classification and immune-related scores are available in the literature (18). The expression levels of these immune genes were quantified as log2 (FPKM + 1). Additionally, we used the ESTIMATE algorithm to calculate the stromal and immune scores and tumor purity of the TCGA-BLCA cohort (24).

### Copy Number Variation Analysis

The Broad GDAC Firehose website (http://gdac.broadinstitute.org/) was used to download the Affymetrix SNP 6.0 microarray data (hg19; germline/potential false-positive calls were removed) from TCGA-BLCA, and the data (CNV segments) were uploaded to the GenePattern (25) web portal (https://cloud.genepattern.org/gp/pages/index.jsf) with Genomic Identification of Significant Targets In Cancer 2.0 (GISTIC 2.0) analysis. GISTIC 2.0 analysis was estimated by default settings, with a confidence level of 0.99 and the removal of data from the X-chromosome before analysis. The R package “Maftools (22)” was used to visualize the CNV analysis results of GISTIC 2.0.

### Gene Set Enrichment Analysis and Analysis of Gene Sets Related to the DDR Pathway

The R package “edgeR” was used to estimate gene expression changes (Raw Count) in the TCGA-BLCA cohort (26). Functional pathway analysis and GSEA were performed with the “clusterProfiler” R package (27) and MSigDB database of the Broad Institute (28). Gene Ontology (GO), Kyoto Encyclopedia of Genes and Genomes (KEGG) and Reactome terms were identified with a cutoff of P <0.05. Enrichment P values were based on 1,000 permutations. The gene sets related to the DDR pathway were collected from MSigDB (28). Detailed data are described in Additional File: **Table S1**. This DDR gene set was used to evaluate the number of non-synonymous mutations in the DDR pathway in the TCGA-BLCA cohort based on NCOR1 alteration status.

### Statistical Analysis

For comparisons of TMB, NAL, immune cell abundance, immune-related gene expression profile, age, MSI score, and mutation counts of the DDR pathway between the NCOR1-WT and NCOR1-MT patients in TCGA were estimated by the Mann–Whitney U test. Differences in the mutation status, sex, sample type and drug type between the NCOR1-WT and NCOR1-MT patients in the ICI-treated cohort were analyzed by Fisher’s exact test. Fisher’s exact test was also used to compare the mutation status, sex, race, ethnicity and clinical stage between the NCOR1-WT and NCOR1-MT patients in the TCGA-BLCA cohort. The KM method was used to generate survival curves for the subgroups in the ICI-treated cohort, and the log-rank test was used to determine the statistical significance of differences. P <0.05 was considered significant, and all statistical tests were two-sided. All statistical tests and visual analyses were completed in R software (version 3.6.1). In addition, the “ggpubr” R package was used to visualize boxplots (29). The false discovery rate (FDR) of CNV visualization was less than 0.05.

## Results

### NCOR1 Mutations Were Linked to Improved OS After ICI Treatment

**Figure 1:**
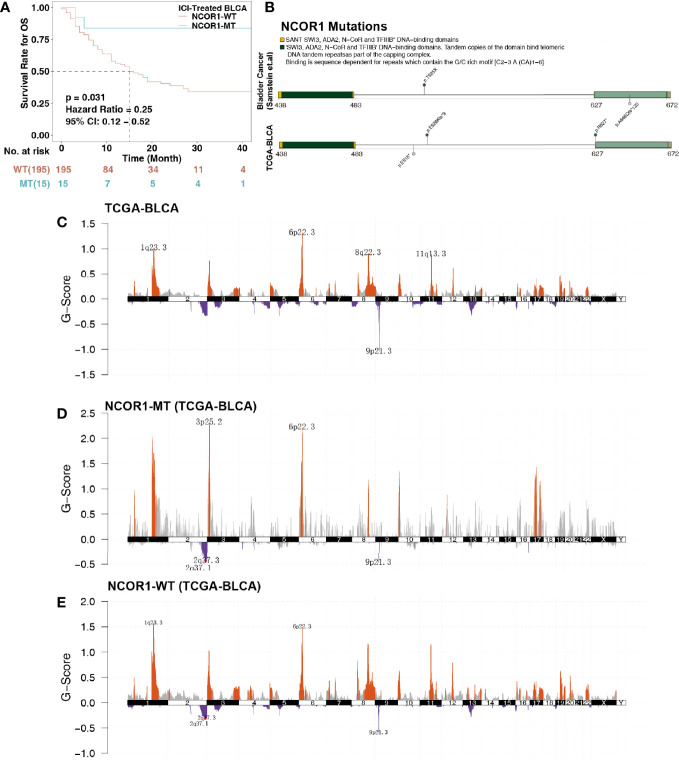
Survival curves and genetic characteristics of patients with BLCA stratified by NCOR1 status. **(A)** Kaplan–Meier estimates of OS in the ICI-treated BLCA cohort comparing patients with NCOR1-MT with their respective counterparts without NCOR1-MT. Patients (BLCA) who harbored NCOR1 mutations showed a better prognosis for ICI-based immunotherapy (P < 0.031, log-rank test). **(B)** Lollipop plot shows the distribution of NCOR1 mutations in the ICI-treated cohort (top panel) and TCGA-BLCA cohort (bottom panel). GISTIC 2.0 analysis showing the most consistent and relevant cytoband alterations in TCGA-BLCA **(C)**, NCOR1-MT **(D)** and NCOR1-WT **(E)**. The annotated cytobands met the criterion of false discovery rate (FDR) ≤0.05. ICIs, Immune checkpoint inhibitors; OS, overall survival; BLCA, bladder urothelial carcinoma; NCOR1, nuclear receptor corepressor 1; TCGA, The Cancer Genome Atlas; MT, mutant; WT, wild-type.

To explore the association between NCOR1 mutations and the efficacy of ICIs in bladder cancer patients, we used cBioportal to download the ICI-treated bladder cancer cohort (Samstein et al.), including 215 patients receiving ICIs (PD-(L)1 and/or CTLA-4 inhibitors. The exclusion criteria for the ICI-treated cohort before further survival analysis were bladder cancer samples with missing follow-up time or missing mutation data (n = 5). Survival analysis showed that the OS of NCOR1-MT patients after ICI treatment was significantly longer than that of NCOR1-WT patients (log-rank test P = 0.031; hazard ratio (HR) = 0.25, 95% CI: 0.12−0.52); median OS (15 months *versus* not reached; **Figure 1A**). Moreover, we found that the TCGA-BLCA patients had prolonged OS times compared with the NCOR1-WT patients in the ICI-treated BLCA cohort (P < 0.001, HR = 0.52, 95% Cl: 0.39–0.7; **Figure S1**). Although there was a significant difference between the two groups, the proportion of stage IV patients of the TCGA-BLCA cohort (33.09%) was also significantly lower than that of the ICI-treated cohort (94%). Thus, the difference between the proportion of stage IV patients may contribute to the difference in the KM analysis. Mutations or deletions of NCOR1 have been described in bladder cancer (30). Thus, deep deletions of NCOR1 have been taken into account. In another ICI-treated cohort (Mariathasan et al.), there were no significant differences in OS between patients with and without whole genomic alteration of NCOR1 (**Figure S2A**). In the TCGA-BLCA cohort, patients with NCOR1-WT had a worse OS compared with NCOR1-DEL patients (P = 0.032, HR = 4.08; 95CI: 2–8.35; **Figure S2B**).

### Mutation and Clinical Characteristics and Chromosomal Analysis Based on NCOR1 Status

NCOR1 is a transcriptional coregulatory protein that includes conserved sequences, such as the DAD, SW13, ADA2, NCOR1, and SANT domains. The interaction between the DAD domain and histone deacetylase 3 (HDAC3) is a necessary condition for the activity of HDAC3. The changed amino acid coordinates of NCOR1 mutations in the ICI-treated and TCGA-BLCA cohorts are shown in **Figure 1B**. We found that NCOR1 somatic mutations (p. R627 * and p. A648Qfs * 120) occurred in the DAD domain. However, NCOR1 mutations did not include any functional hotspot mutations from 3D Hotspots (https://www.3dhotspots.org). Common and significant CNVs in NCOR1-MT and NCOR1-WT patients were identified using GISTIC 2.0. CNV analysis showed that the NCOR1-MT group had significant amplifications at 3p25, and 1q23.3 was identified in only the NCOR1-WT group. The annotated cytobands met the criterion of FDR ≤0.05.

**Figure 2:**
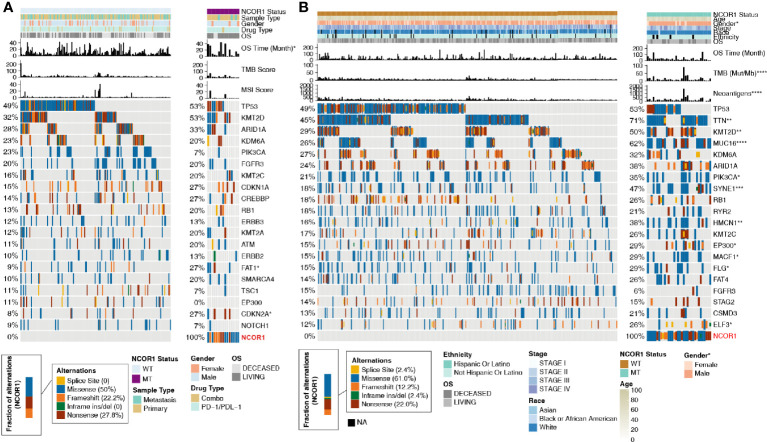
Mutational landscape, clinical information of BLCA patients and the characteristics of the NCOR1 mutations in patients. **(A)** The top 20 frequently mutated genes in BLCA in the Samstein cohort (ICI-treated). The genes were ranked by the mutation frequency in BLCA patients. The asterisks indicate a significant difference between NCOR1-MT and NCOR1-WT. The mutation rates, sex, drug type, and sample type were evaluated using Fisher’s exact test. The TMB and MSI scores were evaluated using the Mann–Whitney U test. **(B)** The top 20 frequently mutated genes in BLCA in the TCGA-BLCA cohort. The genes were ranked by the mutation frequency in BLCA patients. The mutation rates, clinical stage, race, sex and ethnicity were evaluated using Fisher’s exact test. TMB, NAL and age were evaluated using the Mann–Whitney U test. (*P < 0.05, **P < 0.01, ***P < 0.001, and ****P < 0.0001). BLCA, Bladder urothelial carcinoma; NCOR1, nuclear receptor corepressor 1; TMB, tumor mutation burden; NAL, neoantigen load; MT, mutant; WT, wild-type; MSI, microsatellite instability.

Based on the NCOR1 mutation status, the association between the clinical characteristics of patients and the top 20 mutations was analyzed. In the ICI-treated cohort, sex, sample type, drug type, and MSI score were not significantly different between the NCOR1-MT and NCOR1-WT patients. The detailed baseline characteristics of patients with or without an NCOR1 mutation are summarized in **Supplemental Table S2**. In the TCGA-BLCA cohort, compared with the NCOR1-WT group, the proportion of males in the NCOR1-MT group was larger (80.0 *versus* 76.0%, P <0.05). Most of the NCOR1 mutations in the ICI-treated and TCGA-BLCA cohorts were missense mutations (50.0 and 61.0%), nonsense mutations (27.8 and 22.0%) and frameshift mutations (22.2 and 12.2%). The gene mutation profiles of the ICI-treated cohort are summarized in **Figure 2A**, and the results show that FAT1 (27.0 *versus* 9.0%, P <0.05) and CDKN2A (27.0 *versus* 8%, P <0.05) have higher mutation frequencies in NCOR1-MT patients. In the TCGA-BLCA cohort, TTN, KMT2D, MUC16, PIK3CA, SYNE1, HMCN1, EP300, MACF1, FLG and ELF3 had higher mutation frequencies in NCOR1-MT patients.

### Association of NCOR1 Mutations With Enhanced Tumor Immunogenicity, Higher Immune Score and Infiltrated Immune Cells

**Figure 3:**
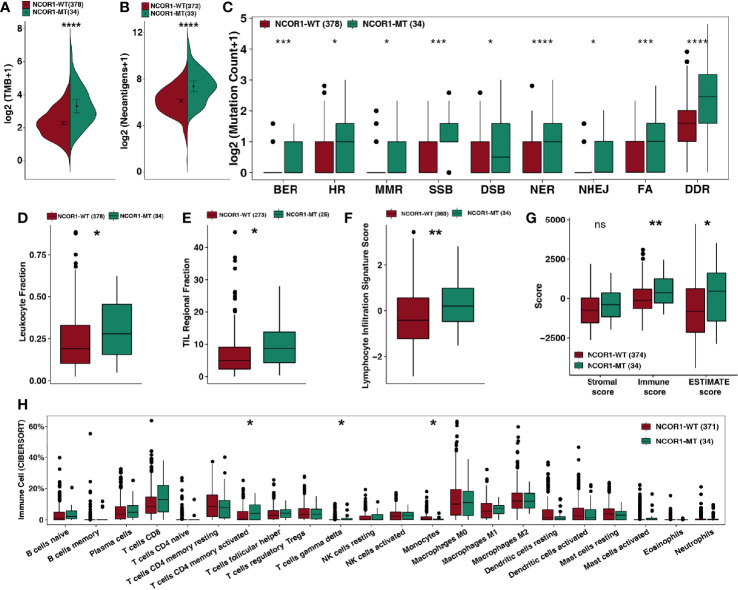
NCOR1 mutations were associated with enhanced tumor immunogenicity, higher immune scores and activated immune cells. **(A, B)** Comparison of TMB **(A)** and NAL **(B)** between NCOR1-MT and NCOR1-WT tumors in the TCGA-BLCA cohort. **(C)** Comparison of DNA damage-related gene set alterations between NCOR1-MT and NCOR1-WT tumors in the TCGA-BLCA cohort. **(D–G)**, Comparison of leukocyte fraction **(D)**, TIL regional fraction **(E)**, lymphocyte infiltration signature score **(F)** and ESTIMATE-derived scores **(G)** between NCOR1-MT and NCOR1-WT tumors in the TCGA-BLCA cohort. **(H)** Comparison of immune cells between NCOR1-MT and NCOR1-WT tumors in the TCGA-BLCA cohort. Gene expression profiles were prepared using standard annotation files, and data were uploaded to the CIBERSORT web portal (http://cibersort.stanford.edu/), with the algorithm run using the LM22 signature and 1,000 permutations. (*P < 0.05, **P < 0.01, ***P < 0.001, and ****P < 0.0001, ns, not significant. **(A–H)** Mann–Whitney U test). TMB, tumor mutation burden; TCGA, The Cancer Genome Atlas; LUAD, lung adenocarcinoma; BLCA, bladder urothelial carcinoma; NCOR1, nuclear receptor corepressor 1; MT, mutant; WT: wild-type.

Based on the results of the current study, enhanced tumor immunogenicity predicts improved clinical response to ICIs. We compared the TMB and NAL between NCOR1-MT and NCOR1-WT tumors (**Figures 3A–C**), and the results showed that TMB [8.0 (4.8–16) Mb/Mut *versus* 3.7 (2.1–6.2) Mb/Mut] and NAL [170.0 (95.0–310.0) Mb *versus* 71.0 (40.0–130.0) Mb] were significantly increased in NCOR1-MT tumors. Based on the number of non-synonymous mutations in the DDR pathway related to the efficacy of ICIs (31–33), we used a DDR gene set (**Supplementary Table**) to compare the difference in the number of mutations in the DDR pathway between NCOR1-MT and NCOR1-WT tumors. **Figure 3C** shows that NCOR1-MT tumors had a significantly increased number of DDR pathway mutations (such as BER, HR, MMR, SSB, DSB, NER, and NHEJ, all P < 0.05). Additionally, the NCOR1-MT group had significantly higher TMB, NAL and DDR mutations compared with NCOR1-depletion (NCOR1-DEL) or no-alteration group (**Figure S3**).

The above results suggest that the genome in NCOR1-MT tumors may be more unstable and may more easily accumulate mutations.

The TME is one of the important factors affecting the efficacy of ICIs and includes indicators such as tumor-infiltrating lymphocytes (TILs), the immune score, the stromal score and the cytolytic (CYT) score. Based on the NCOR1 mutation status, we compared the immune-related score (**Figures 3D–F**) calculated by Thorsson et al. The results showed that NCOR1-MT tumors had a higher leukocyte fraction score [0.28 (0.16–0.46) *versus* 0.19 (0.1–0.33); P <0.05], TIL regional fraction score [8.7 (4.3–14) *versus* 4.9 (2.3–9.1); P <0.05] and leukocyte infiltration signature score [0.2 (−0.47–0.98) *versus* −0.41 (−1.2–0.56); P < 0.01]. Similarly, the results of the ESTIMATE algorithm suggested that NCOR1-MT tumors have a higher immune score [370 (−290–1,200) *versus* −120 (−650–580); P < 0.01) and tumor purity [460 (−1400–1600) *versus* −810 (−2,100–620); P < 0.05]. The analysis results based on the CIBERSORT algorithm showed that activated CD4+ memory T cells and gamma delta T cells were highly infiltrated in NCOR1-MT tumors (all P < 0.05).

### NCOR1 Mutations and Immune GEPs

**Figure 4:**
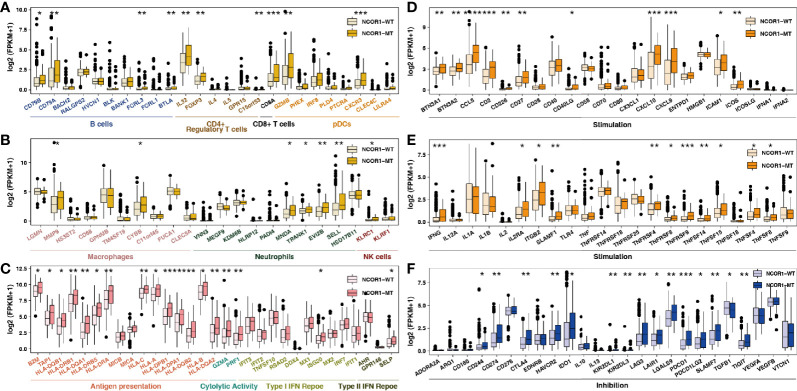
NCOR1 mutations were associated with activated antitumor immunity. The expression levels of immune-related genes, such as immune cells **(A, B)**, antigen presentation-related genes, cytolytic activity-associated genes, IFN-response genes **(C)**, stimulation-related genes **(D, E)** and inhibition-related genes **(F)** in NCOR1-MT tumors *versus* NCOR1-WT tumors in TCGA-BLCA. (*P < 0.05, **P < 0.01, ***P < 0.001, and ****P < 0.0001, (**A**–**F**: Mann–Whitney U test). TCGA, The Cancer Genome Atlas; LUAD, lung adenocarcinoma; BLCA, bladder urothelial carcinoma; NCOR1, nuclear receptor corepressor 1; MT, mutant; WT, wild-type.

Studies suggest that specific GEPs are related to the clinical response to ICIs. According to the classification of immune gene function by Rooney et al. (34) and Thorsson et al., we compared the differences between immune-related GEPs in NCOR1-MT and NCOR1-WT tumors. NCOR1-MT tumors were associated with significantly upregulated expression of genes related to activated immune cells (such as B cells, CD4+ regulatory T cells, CD8+ T cells, macrophages, neutrophils and NK cells; **Figures 4A, B**). NCOR1-MT tumors had higher expression of antigen presentation-, cytolytic activity- and IFN response-related genes (**Figure 4C**). The results of the analysis of stimulating immune-related genes (**Figures 4D, E**) showed that chemokines, cytokines, and tumor necrosis factor receptor superfamily (TNFRSF)-related genes, including the well-known CXCL9, CXCL10, and IFNG genes (all P < 0.05), were significantly upregulated in NCOR1-MT tumors. NCOR1-MT tumors had significantly increased expression levels of immune checkpoint-related genes, such as CD274, CTLA4, LAG3, PDCD1, PDCD1LG2, and TIGIT (**Figure 4F**; P < 0.05). In addition, NCOR1-MT tumors had significantly increased expression levels of immune-depletion-related genes, such as HAVCR2 and IDO1 (**Figure 4F**; all P < 0.05).

### Transcriptome Traits Based on NCOR1 Status

**Figure 5:**
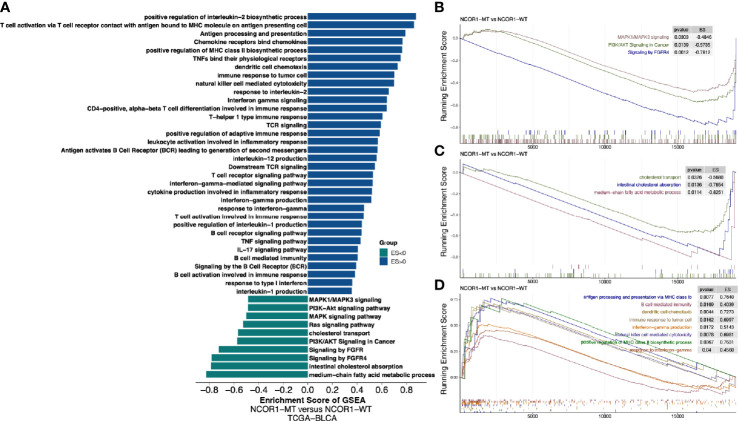
Transcriptome biological function traits of NCOR1-MT and NCOR1-WT tumors in the TCGA-BLCA cohort. **(A)** Differences in pathway activities scored by GSEA between NCOR1-MT and NCOR1-WT tumors in the TCGA-BLCA cohort. The enrichment results with significant associations between NCOR1-MT and NCOR1-WT tumors are shown. The blue bar indicates that the ES of the pathway is more than 0, while the green bar indicates that the ES of the pathway is less than 0. **(B–D)** GSEA of the hallmark gene sets downloaded from MSigDB. All transcripts were ranked by the log2 (fold change) between NCOR1-MT and NCOR1-WT tumors in the TCGA-BLCA cohort. Each run was performed with 1,000 permutations. The enrichment results with significant associations between NCOR1-MT and NCOR1-WT tumors are shown. NCOR1, nuclear receptor corepressor 1; MT, mutant; WT, wild-type; ES, enrichment score; GSEA, gene-set enrichment analysis.

Further analysis of the differences in the potential biological mechanisms between NCOR1-MT and NCOR1-WT tumors and the results of GSEA (**Figures 5A–D**) showed that oncogenic signaling pathways, such as the MAPK1/MAPK3, PI3K/AKT, and FGFR4 pathways, were significantly downregulated in the NCOR1-MT group. Similarly, metabolic-related pathways, such as cholesterol ester transport, intestinal cholesterol absorption, and medium-chain fatty acid metabolic process, were significantly downregulated in the NCOR1-MT group (**Figures 5A, C**). In contrast, immune response-related pathways, such as antigen processing and presentation *via* MHC class Ib, positive regulation of MHC class II biosynthetic process, dendritic cell chemotaxis, immune response to tumor cells, natural killer cell-mediated cytotoxicity, interferon-gamma production, response to interferon-gamma, and B cell-mediated immunity, were significantly upregulated in the NCOR1-MT group (**Figures 5A, D**).

## Discussion

**Figure 6:**
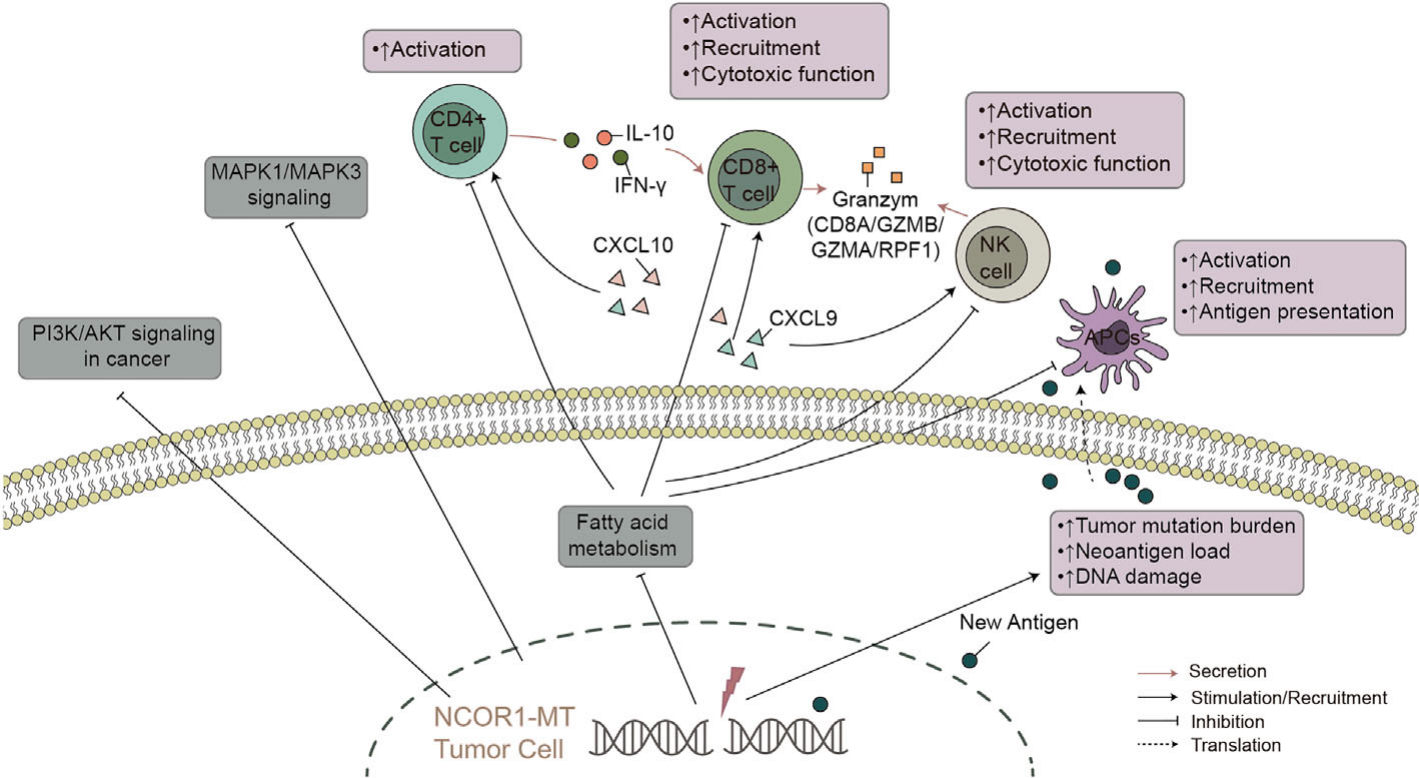
The possible mechanism underlying the improved efficacy and prognosis in NCOR1-MT BLCA receiving ICIs. NCOR1, nuclear receptor corepressor 1; MT, mutant; BLCA, bladder urothelial carcinoma; ICIs, immune checkpoint inhibitors.

Bladder cancer is one of the most common urinary system cancers worldwide, but traditional treatments have not significantly improved the 5-year survival rate (3). In recent years, immunotherapy (especially targeted PD-(L)1 and/or CTLA-4) has shown excellent prospects in bladder cancer. However, most patients with bladder cancer do not have an effective antitumor immune response, and guidance for immunotherapy lacks precise biomarkers. In this study, we found that compared with NCOR1-WT patients, NCOR1-MT patients have a significantly better OS. Based on the NCOR1 mutation status, we explored possible factors that may affect the difference in immunotherapy efficacy in tumor patients. We aimed to illustrate the interactions between NCOR1 mutations and the mechanisms of improved response to ICIs. We found some possible mechanisms that may help to improve clinical outcomes in NCOR1-MT bladder cancer (**Figure 6**): higher tumor immunogenicity (such as TMB, NAL, the number of DDR pathway mutations and the upregulated expression of antigen presentation-related genes); highly infiltrating TILs in the TME (such as CD4 + T cells and delta gamma T cells); upregulated expression of immune-related genes (such as immune-cell inflamed-related genes, IFN-*γ*, CYT, immune checkpoints and chemokines); and higher immune scores (such as TIL regional fraction score, leukocyte fraction score and leukocyte infiltration signature score). In addition, the GSEA results suggested that immune response-related pathways were significantly enriched in NCOR1-MT tumors. In contrast, oncogenic signaling pathways and metabolic-related pathways were significantly downregulated in NCOR1-MT tumors. Overall, these data indicate an association between NCOR1 mutations and improved survival after ICIs.

Specific genetic mutations have been determined to predict the response to ICB therapy. For instance, mutations in the interferon signaling pathway genes JAK1/2, B 2M, IFNGR1, IFNGR2, and APLNR indicate that patients with melanoma are resistant to immunotherapy (35, 36); mutations in genes related to the DDR pathway, such as POLE, POLD1, MLH1, MSH2, PMS2, MSH6, BRCA1, BRCA2, TP53, PRKDC, LIG3, RAD17, RAD51C, FANCF, and ERCC1, are associated with higher TMB and improved response to ICIs (37, 38).

Regarding the regulation of the immune response, it has been reported that NCOR1 plays an important role in immune tolerance and the development of T cells. In previous studies, NCOR1 depletion upregulated a wide variety of tolerogenic genes in activated DCs ex vivo and *in vivo*, which consequently resulted in an increased frequency of FoxP3+ Tregs (15). Furthermore, a previous study suggested that NCOR1-HDAC complexes can inhibit the enhancing activity of Tregs (14). Therefore, we explored various mechanisms of the response to ICIs based on NCOR1 status in bladder cancer, which may serve as a future potential biomarker. NCOR1-MT tumors were related to longer OS in bladder cancer patients after ICI treatment. Then, we investigated certain factors predicting the response to ICIs, such as tumor immunogenicity, immune-related scores, immune cells, immune-related GEPs and the DDR pathway.

Multiple studies have demonstrated that tumor immunogenicity results in a promising response to ICIs (39–41). For example, high TMB has predictive value in a variety of cancers (40, 41). In addition, genome somatic mutations may produce new antigenic peptides. It is generally believed that tumors carrying more mutations may produce more new epitopes that are recognized by TILs. High NAL tumors could be more effective for ICIs, which has been shown in melanoma and non-small cell lung cancer (NSCLC) (39). DDR pathway mutations mediate genomic variation, high heterogeneity, and instability in tumors, which has been emphasized in ICIs. For example, tumors with mutations in the DDR pathway can accumulate a large amount of uncorrected DNA damage, leading to high TMB and independently relating to the efficacy of ICIs (31, 32). Hypothetically, the immunogenicity of tumors will also be affected by components related to the TME, such as the efficiency of antigen processing and presentation of professional antigen-presenting cells (pAPCs) (42). MHC-II molecules are mainly expressed by pAPCs such as DCs, B cells, and macrophages. Raw peptide antigens derived from foreign sources are presented to CD4+ T cells by MHC-II molecules. The expression of tumor-specific MHC-II molecules is associated with enhancing the ICI treatment response (43). Overall, higher immunogenicity (TMB, NAL, DDR pathway mutations and the expression of antigen presentation-related genes) may be an underlying mechanism related to NCOR1-MT tumors and promising responses to ICIs.

In addition to tumor immunogenicity, the TME, including TILs, chemokines, and cytokines, is also one of the most important factors affecting the efficacy of ICIs. TILs are a group of tumor-infiltrating and antigenic cell groups (such as T cells, B cells, NK cells, macrophages and DCs) that can exist in tumor nests and between tumors. Multiple studies have demonstrated that highly infiltrating TILs (such as CD8+ T cells and CD4+ T cells) are associated with improved ICI efficacy in a variety of tumors (3, 4, 6). In addition, specific chemokines play a vital role in recruiting immune cells to enhance antitumor immunogenicity. Notably, the CXCL9/CXCL10/CXCL11/CXCR3 axis recruits cytotoxic lymphocytes, NK cells, NKT cells, and macrophages to infiltrate tumor tissue and increases cytotoxic activity against tumor cells (44, 45). IFN-*γ* not only recruits immune cells to initiate antitumor proliferation and cause tumor apoptosis (46) but also mediates CD8+ T cells to promote iron death in tumor cells (47). Collectively, these data indicate that highly infiltrated TILs (CD4 + memory T cells) and the upregulated expression of immune cell-related genes (such as B cells, CD4 + regulatory T cells, CD8 + T cells, macrophages, neutrophils and NK cells), chemokines (CXCL9/CXCL10) and cytokines (IFN-*γ*) may improve the immune response in NCOR1-MT cells.

Recent studies suggest that T-cell inflamed and IFN-*γ*-related GEPs resulted in efficacy against ICIs (48). For example, the increased expression of CD8A, CD8B, GZMA, GZMB, and PRF1 is a predictor of higher CYT and better immune therapy response (48). Additionally, the upregulated expression of immune checkpoints is positively associated with response to ICIs (49, 50). Ahad et al. found that NCOR1 directly represses the tolerogenic program in conventional dendritic cells (cDCs), inhibiting the transcription of tolerogenic genes (*e.g.*, IL-10, IDO, TGFBR1, IL-27 and PD-L1). In contrast, the depletion of NCoR1 in cDCs can lead to the development of Tregs, and cDCs can eventually become tolerogenic DCs. Consistent with the findings of Ahad et al., we found that patients with NCOR1-MT have a higher expression of immune depletion-related genes (such as CD274, CTLA4, LAG3, PDCD1, PDCD1LG2, TIGIT and IDO1).

The GSEA results also suggested that patients with NCOR1-MT have a better prognosis after ICI treatment. For example, immune-related response pathways, such as antigen presentation, IFN-γ production, and NK-mediated cytotoxic activity against tumors, were significantly upregulated in NCOR1-MT patients. There is a growing body of evidence demonstrating that the transportation and uptake of fatty acids can promote cancer metastasis and progression (51). Notably, the combination of cholesterol with the TCRβ transmembrane region can inhibit the cytotoxic activity of T cells against tumors (52). Our data are also consistent with the hypothesis that NCOR1-MT is associated with the development of an antitumor immune response (51–53).

This study analyzed the prognosis of ICI treatment and NCOR1 mutations in patients with bladder cancer and attempted to elucidate the underlying mechanism of NCOR1-MT as a biomarker for screening the predominant population of bladder cancer preferred for immunotherapy; however, there are still some limitations. First, this study included only one ICI-treated cohort of bladder cancer, which may introduce bias when screening biomarkers for the prognosis of ICIs of bladder cancer. Second, targeted sequencing (MSK-IMPACT) was used to detect somatic mutations in the ICI-treated cohort and included significantly fewer gene mutations than does whole-exome sequencing (WES). Third, there is currently no direct evidence of NCOR1 transcriptomics, CNV and protein levels and other data related to the prognosis of ICI treatment of bladder cancer patients. Fourth, there were some co-occurrence/mutually exclusive genes accompanying NCOR1 mutations. We hope to conduct relevant cell or animal experiments in the future to verify how NCOR1 mutations at different sites affect the efficacy of immunotherapy, explore the relationship between NCOR1 mutations and the TME, provide typical cases to further support this hypothesis and determine the role of other mutated genes that accompany NCOR1 mutations.

## Conclusion

NCOR1 mutations may be a potential biomarker for predicting the prognosis of bladder cancer patients undergoing ICI treatment. In addition to prolonged OS after ICIs, NCOR1-MT positively correlated with many known predictive biomarkers of immunotherapy, including TMB, NAL, immune-related GEPs, immune cells and DDR pathway mutations. This result has translational relevance by providing a comprehensive profile of NCOR1 status in bladder cancer and a basis for further biomarker studies targeting NCOR1.

## Data Availability Statement

The original contributions presented in the study are included in the article/**Supplementary Material**. Further inquiries can be directed to the corresponding authors.

## Author Contributions

AL wrote the manuscript. PL and JZ designed the research. AL and ZQ performed the research. AL, ZQ, PL, and JZ, Writing—review and editing. All authors contributed to the article and approved the submitted version.

## Funding

This work was supported by grants from the Science and Technology Project of Education Department of Jiangxi Province (Number 180812) and the Science and Technology Project of Health and Family Planning Commission of Jiangxi Province (Number 20195361).

## Conflict of Interest

The authors declare that the research was conducted in the absence of any commercial or financial relationships that could be construed as a potential conflict of interest.

## Supplementary Material

Supplementary Table 2 The detailed baseline characteristics of patients with or without NCOR1 mutations.

Supplementary Figure 1 Kaplan–Meier estimates of OS in the ICI-treated BLCA cohort and TCGA-BLCA cohort.

Supplementary Figure 2 Kaplan–Meier estimates of OS in the ICI-treated BLCA cohort (Mariathasan et al; **(A)** and TCGA-BLCA cohort **(B)**.

Supplementary Figure 3 Differences in TMB, NAL, or the counts of DDR mutations between the NCOR1-MT, NCOR1-DEL and no-alteration groups.

## Abbreviation

ICB, Immune checkpoint blockade; BLCA, bladder cancer; OS, overall survival; NCOR1-MT, NCOR1-mutant; NCOR1-WT, NCOR1-wild-type; anti-PD-(L)1, anti-programmed cell death (ligand) 1; anti-CTLA-4, anti-cytotoxic T lymphocyte-associated antigen 4; ICIs, immune checkpoint inhibitors; TMB, tumor mutational burden; GEPs, gene expression profiles; DDR, DNA damage response; NCOR1, nuclear receptor corepressor 1; DCs, dendritic cells; Tregs, regulatory T cells; NCOR1-HDAC, NCOR1-histone deacetylase; KM, Kaplan–Meier; NGS, next-generation sequencing; NAL, neoantigen load; TCGA-BLCA, The Cancer Genome Atlas Urothelial Bladder Carcinoma; GISTIC, Genomic Identification of Significant Targets In Cancer; GSEA, Gene set enrichment analysis; GO, Gene Ontology; KEGG, Kyoto Encyclopedia of Genes and Genomes; FDR, false discovery rate; HDAC3, histone deacetylase 3; TILs, tumor-infiltrating lymphocytes; CYT, cytolytic; TNFRSF, tumor necrosis factor receptor superfamily; NSCLC, non-small cell lung cancer; pAPCs, professional antigen presenting cells; cDCs, conventional dendritic cells; WES, whole-exome sequencing.
