# Supplementary material for: Effect of NCOR1 Mutations on Immune Microenvironment and Efficacy of Immune Checkpoint Inhibitors in Patient with Bladder Cancer
Source: Front Immunol. 2021 Mar 8;12:630773. doi: 10.3389/fimmu.2021.630773 (PMC7982737; doi:10.3389/fimmu.2021.630773)
Supplement: Supplementary Table 2 — The detailed baseline characteristics of patients with or without NCOR1 mutations. [file Table_2.pdf]

	NCOR1-MT (N=15)	NCOR1-WT (N=195)	Overall (N=210)
**MSI_score**			
Mean (SD)	2.60 (6.39)	1.40 (4.44)	1.48 (4.60)
Median [Min, Max]	0.300 [0, 22.7]	0.320 [0, 41.3]	0.315 [0, 41.3]
**Age**			
Mean (SD)	67.8 (14.7)	67.1 (10.0)	67.1 (10.4)
Median [Min, Max]	71.0 [32.0, 89.0]	68.0 [39.0, 86.0]	68.0 [32.0, 89.0]
**TMB_Score**			
Mean (SD)	19.6 (18.3)	12.9 (17.9)	13.4 (18.0)
Median [Min, Max]	14.9 [2.95, 71.8]	7.90 [0.878, 210]	8.78 [0.878, 210]
**Gender**			
Female	3 (20.0%)	48 (24.6%)	51 (24.3%)
Male	12 (80.0%)	147 (75.4%)	159 (75.7%)
**Drug_Type**			
Combo	1 (6.7%)	22 (11.3%)	23 (11.0%)
PD-1/PDL-1	14 (93.3%)	173 (88.7%)	187 (89.0%)
**Sample_Type**			
Metastasis	3 (20.0%)	86 (44.1%)	89 (42.4%)
Primary	12 (80.0%)	109 (55.9%)	121 (57.6%)
