# Supplementary figures and images for: Effect of NCOR1 Mutations on Immune Microenvironment and Efficacy of Immune Checkpoint Inhibitors in Patient with Bladder Cancer
Source: Front Immunol. 2021 Mar 8;12:630773. doi: 10.3389/fimmu.2021.630773 (PMC7982737; doi:10.3389/fimmu.2021.630773)

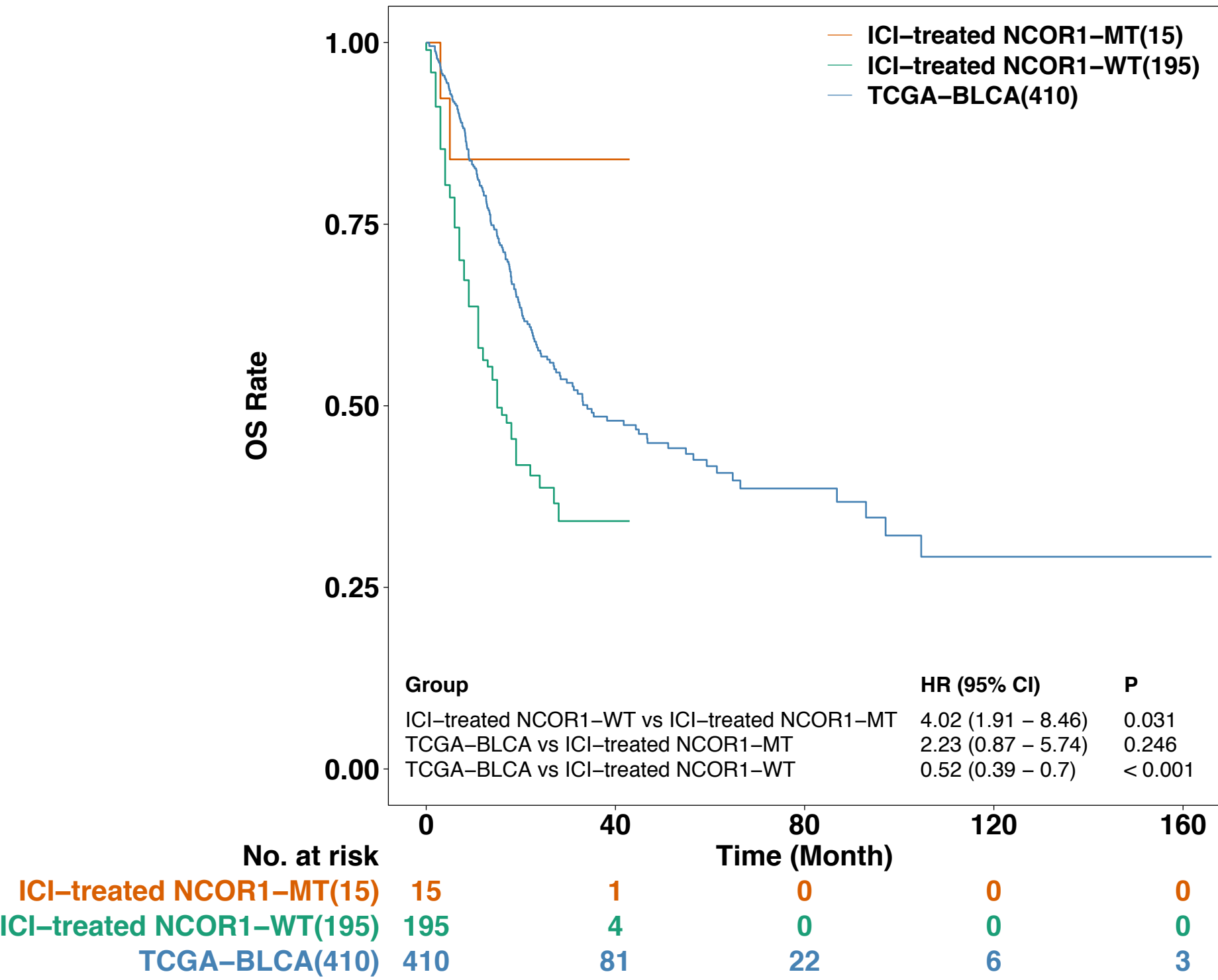

Supplement: Supplementary Figure 1 — Kaplan–Meier estimates of OS in the ICI-treated BLCA cohort and TCGA-BLCA cohort. [file Image_1.pdf]

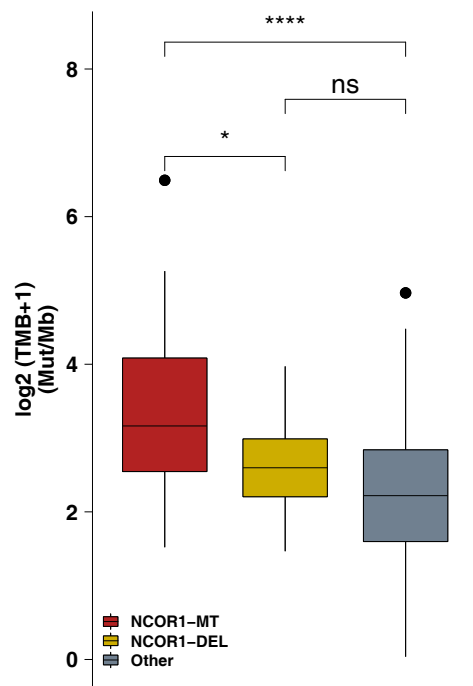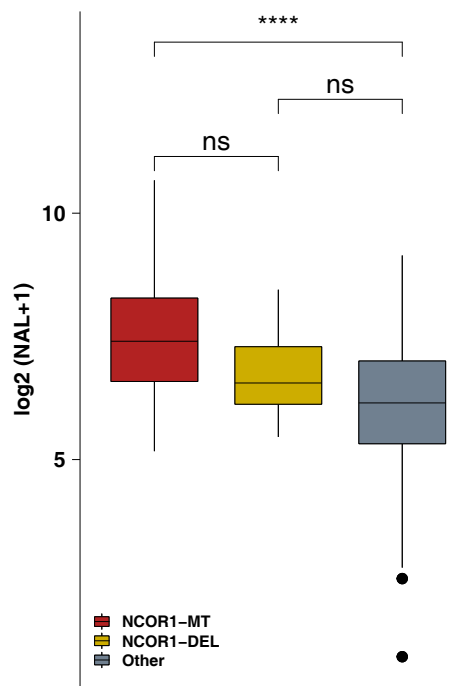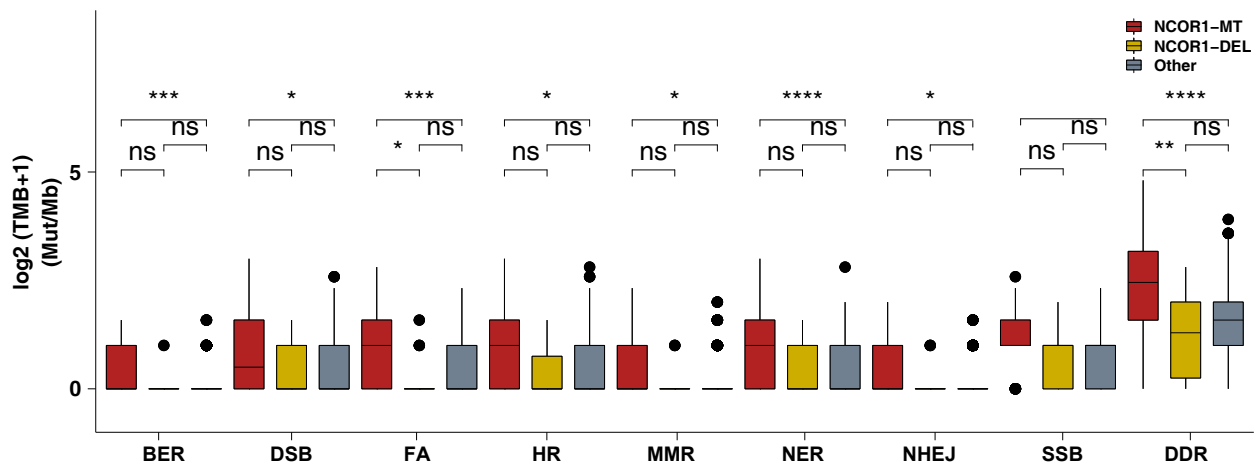

Supplement: Supplementary Figure 3 — Differences in TMB, NAL, or the counts of DDR mutations between the NCOR1-MT, NCOR1-DEL and no-alteration groups. [file Image_3.pdf]
