# Supplementary material for: Effect of NCOR1 Mutations on Immune Microenvironment and Efficacy of Immune Checkpoint Inhibitors in Patient with Bladder Cancer
Source: Front Immunol. 2021 Mar 8;12:630773. doi: 10.3389/fimmu.2021.630773 (PMC7982737; doi:10.3389/fimmu.2021.630773)
Supplement: Supplementary Figure 2 — Kaplan–Meier estimates of OS in the ICI-treated BLCA cohort (Mariathasan et al; (A) and TCGA-BLCA cohort (B). [file Image_2.pdf]

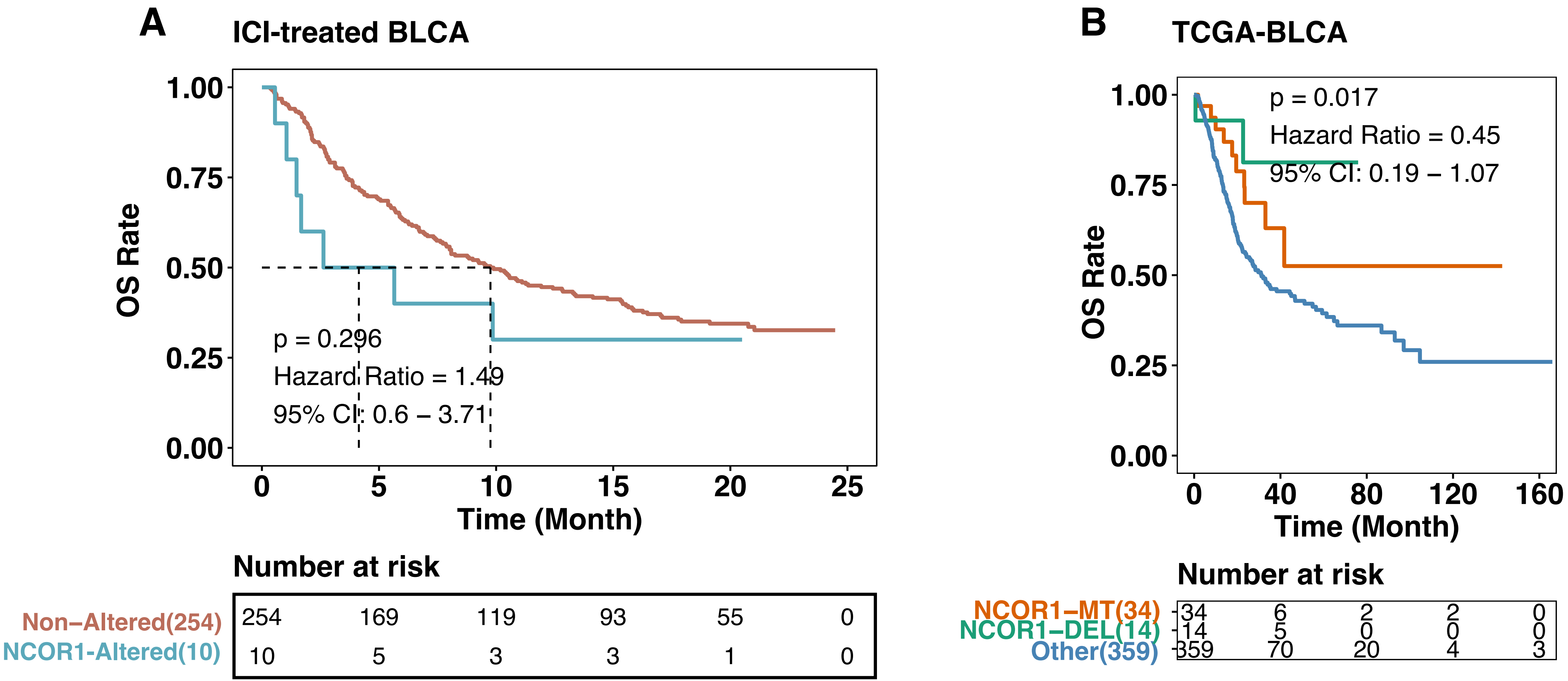
A
ICI-treated BLCA
OS Rate
1.00
0.75
0.50
0.25
0.00
p = 0.296
Hazard Ratio = 1.49
95% CI: 0.6 – 3.71
0
5
10
15
20
25
Time (Month)
Number at risk
Non−Altered(254)
NCOR1-Altered(10)
254 169 119 93 55 0
10 5 3 3 1 0
B
TCGA-BLCA
OS Rate
1.00
0.75
0.50
0.25
0.00
p = 0.017
Hazard Ratio = 0.45
95% CI: 0.19 – 1.07
0
40
80
120
160
Time (Month)
Number at risk
NCOR1−MT(34)
NCOR1−DEL(14)
Other(359)
34 6 2 2 0
14 5 0 0 0
359 70 20 4 3
